# Knowledge, attitudes, and practices regarding immunity boosting dietary behavior of mass population amid COVID-19

**DOI:** 10.1371/journal.pgph.0001872

**Published:** 2023-05-03

**Authors:** Mehedy Hasan Abir, Mahdi Al Hasan Rahat, Silvia Naznin Etu, Tahmid Hussain, Anik Chakraborty, Mahabub Alam, Emily Litzow, Mohammad Mahmudul Hassan

**Affiliations:** 1 Faculty of Food Science and Technology, Chattogram Veterinary and Animal Sciences University, Chattogram, Bangladesh; 2 Faculty of Biological Sciences, Department of Genetic Engineering and Biotechnology, University of Chittagong, Chittagong, Bangladesh; 3 Faculty of Veterinary Medicine, Chattogram Veterinary and Animal Sciences University, Chattogram, Bangladesh; 4 Queensland Alliance for One Health Sciences, School of Veterinary Sciences, The University of Queensland, Gatton, Queensland, Australia; PLOS: Public Library of Science, UNITED STATES

## Abstract

During the increasing spread of COVID-19 occurrences in Chattogram Metropolitan Area (CMA) of Bangladesh, a series of measures were taken to control the transmission. These measures greatly influenced the knowledge, attitudes, and practices (KAP) of the population on their dietary behavior. However, there are no current studies demonstrating the KAP of the CMA citizens regarding their dietary habit that can boost the immunity. In this study, we appraised KAP in regard to immunity boosting dietary behavior from April 26, 2021 to November 17, 2021 during implementation of lockdown measures by the government of Bangladesh. Apart from the basic knowledge and attitudes toward immunity boosting dietary behavior, we have also aimed to assess the practices of the population by whether the nutrients, especially vitamin A, B_6_, B_9_, B_12_, C, D, E, and trace minerals such as zinc, selenium, and iron were included in their diet and in what frequency. This study is a cross-sectional study, and the participants were recruited using both online platforms during the lockdown and through in-person interviews after the withdrawal of lockdown. After obtaining the proper consent from the participants, their sociodemographic variables, and KAP towards immunity boosting dietary behavior were assessed. Total 400 participants were included in this study and a non-probability sampling technique named purposive sampling has been followed for participants recruitment. Among the 400 participants, the majority of them (64.3%) were male, most of them (62.7%) were students, unmarried (69.5%), aged between 18–35 years (82.5%), had a bachelor’s degree (50.0%), and had a monthly family income between 10000–30000 BDT (35.5%). This study indicated that 82.8% of the populations had the correct knowledge, 71.3% had favorable attitudes, and 44% had good practices regarding immunity boosting diet during COVID-19. The majority (79.3%) of the participants had an idea about nutrition, most of them (78.5%) knew the nutrients needed to strengthen their immune system, almost all (98.5%) washed fruits and vegetables purchased from the market before eating them, 78% did not often purchase food online, and 53% often ate junk food. In a binary logistic regression, correct knowledge was significantly associated with the females, having HSC or bachelor’s degree, being in the occupation of business, laborer or others, and having a monthly family income between 50000–100000 or >100000. The favorable attitudes were significantly associated with having a master’s degree or above, and for government job holders. However, the good practices did not show any significant association with the sociodemographic factors in binary logistic regression. Moreover, the study found the presence of bad or unhealthy practices among the populations despite having correct knowledge and favorable attitudes. Thus, this study could identify the variables, such as gender differences, education, monthly family income, and occupation on which emphasis should be given during public health campaigns or training programs to improve the KAP regarding immunity boosting diet.

## 1. Introduction

In December 2019, a pneumonia-like outbreak in China’s Wuhan City of Hubei Province caused disquietude among researchers and medical professionals. Later, it came to light that this outbreak was caused by a newly identified coronavirus strain named SARS-CoV-2 [1]. This discovery led to a strict lockdown situation and disruption of everyday life, including dietary habit and physical activity. Millions of people around the world were infected and killed by this virus. However, the World Health Organization (WHO) suggested that a healthy diet can bolster people’s immunity, and be influential in the prevention and treatment of the disease [2]. Later, several recommendations on dietary intake and guidelines regarding healthy eating were provided for the people confined at home, because the quality of people’s food and their health are closely related [3]. Buying and selling of fresh food became quite difficult because of the lockdown, which affected the supply chain of fresh produce and changed people’s purchasing habits. In addition, the COVID pandemic caused noticeable increases in social discrepancies, where the poorest people suffered the most [4].

Among the various management approaches of COVID-19, healthy dietary behavior, nutrition, lifestyle and environment proved to significantly boost immunity and assisted in maintaining good health [5,6]. A diverse and balanced diet can greatly ameliorate the immune responses to the COVID-19 infection [7], and the healthy foods have been proved to function as a promising therapeutic agent to boost the immunity and to recuperate from the acute respiratory illness and subsequent health consequences, which may promote the protection of mass population during the global pandemic [8]. Some groups of foods, such as fish and fish-products, meat and meat products, fruits and vegetables etc. showed significant advantage against viral infection, including the defense against COVID-19 [9]. Some of the nutrients, including vitamin A, B_6_, B_9_, B_12_, C, D, E, and trace minerals such as zinc, selenium, and iron increase the body’s capability to fight against viral infections [10,11]. Relevant scientific studies showed that sufficient intake of these vitamins and minerals can decrease the inflammatory and oxidative stresses, thus bolstering the immunity of the individuals [12,13].

The COVID-19 inducing home quarantine measures were suggested in multiple cities in various countries, including Chattogram of Bangladesh [14]. The lockdown was required for the protection of the mass population from viral infection [15]. Despite the lockdown manifesting undesirable repercussions, such as dietary behavior changes [16] and psychological stresses [17], healthy eating patterns showed greater benefit in recovering from these repercussions [18]. The people who consumed foods containing beneficial nutrients, generally showed less risk of anxiety or depression [19].The infection characteristics of the virus also showed that adults over 65 years old were at greater risk for infection compared to those under 65. Therefore, the sub-optimal lifestyle of the population associated with their age needs to be studied carefully [20,21].

It is evident that in light of the current global pandemic situation, where people are in distress due to quarantine, it is important to perform substantive research into the influence of COVID-19 on the regular diet, and ways of combating COVID-19 with a diet that bolsters people’s immunity. Studying this will assist epidemiologists, public health researchers and dietitians in outlining their guidelines and dietary recommendations for future pandemics [21].

Knowledge, attitudes, and practices can play vital role in shaping individuals’ dietary behaviors and their impact on immunity. A KAP study in Iran among Tehranian adolescents showed the role of healthy nutrition in preventing the non-communicable diseases [22]. Previous studies in Bangladesh have demonstrated the KAP towards dietary salt intake among nurses of Bangladesh [23], determinants of nutrition KAP on adolescent sports trainee in Bangladesh [24], and balanced diet related KAP among adolescent school girls of Bangladesh [25]. However, currently there is no KAP study regarding immunity boosting dietary behavior amid COVID-19 in Bangladesh, let alone in CMA.

Therefore, the objective of this study is to determine the knowledge, attitudes, and practices regarding immunity boosting dietary behavior along with their occupational differences during COVID-19 among the citizens of CMA. The study was formulated and conducted through an online survey as well as through in-person interviews. Lifestyles during quarantine and the dietary behaviors were taken into consideration for better comparison and comprehension of the practices among the citizens of CMA, as well as to examine the possible risks of nutrient insufficiencies.

## 2. Materials and methods

### 2.1. Ethical statement

This study has been carried out in accordance with the WMA Declaration of Helsinki–Ethical Principles for Medical Research Involving Human Subjects 1964. Formal ethical approval was granted by the Ethics Committee of the Chattogram Veterinary and Animal Sciences University, Bangladesh (Ethical Committee permit reference number: CVASU/Dir (R and E) EC/2019/126 (02), Date: 29 December 2019). The nature of this study was voluntary, and consent from each participant (verbal and written) was taken before inclusion in the study. The information of all the participants were kept anonymous, and confidentiality was properly maintained.

### 2.2 Participant recruitment and study design

A cross-sectional study was conducted among general public during the period of April 26, 2021 to November 17, 2021, and the data were collected online when the government of Bangladesh implemented lockdown strategy. Data were also collected through offline interviews after the withdrawal of lockdown. We have collected data through a non-probability sampling technique named purposive sampling. Half of our samples was collected online and the remaining half of the samples were collected through in-person interviews. Initially, a pre-structured questionnaire was developed using Google Forms in English language while providing the Bengali translation in the parenthesis. We pretested the questionnaire before final administration to the study population. The web-link of the questionnaires were then distributed to the participants through various social media platforms (Facebook Messenger, WhatsApp etc.). The investigators’ idea for collecting the data through online platforms was to maintain social distance amid the confinement situation of Bangladesh. However, when the situation of COVID-19 improved and the lockdown was withdrawn, the investigators went into the field and collected data through in-person interviews from several regions of Chattogram, Bangladesh to include all categories of people in the survey. The in-person interview was conducted in the native language, Bengali. Cochran’s sample size formula for large populations has been used for the calculation of sample size [26] with 95% confidence interval, 5% margin of error, and an assumption that 50% of the respondents having immunity boosting dietary behavior. The formula is as follows:

$$n_{0}=\frac{Z^{2}pq}{e^{2}}$$

Here, n_0_ = Sample size; Z = Z-score = 1.96 for 95% level of confidence; p = Estimated proportion of the attribute that is present in the population = 50% (or 0.5); q = 1-p = 0.5; e = Margin of error = 5%.

**Fig 1:**
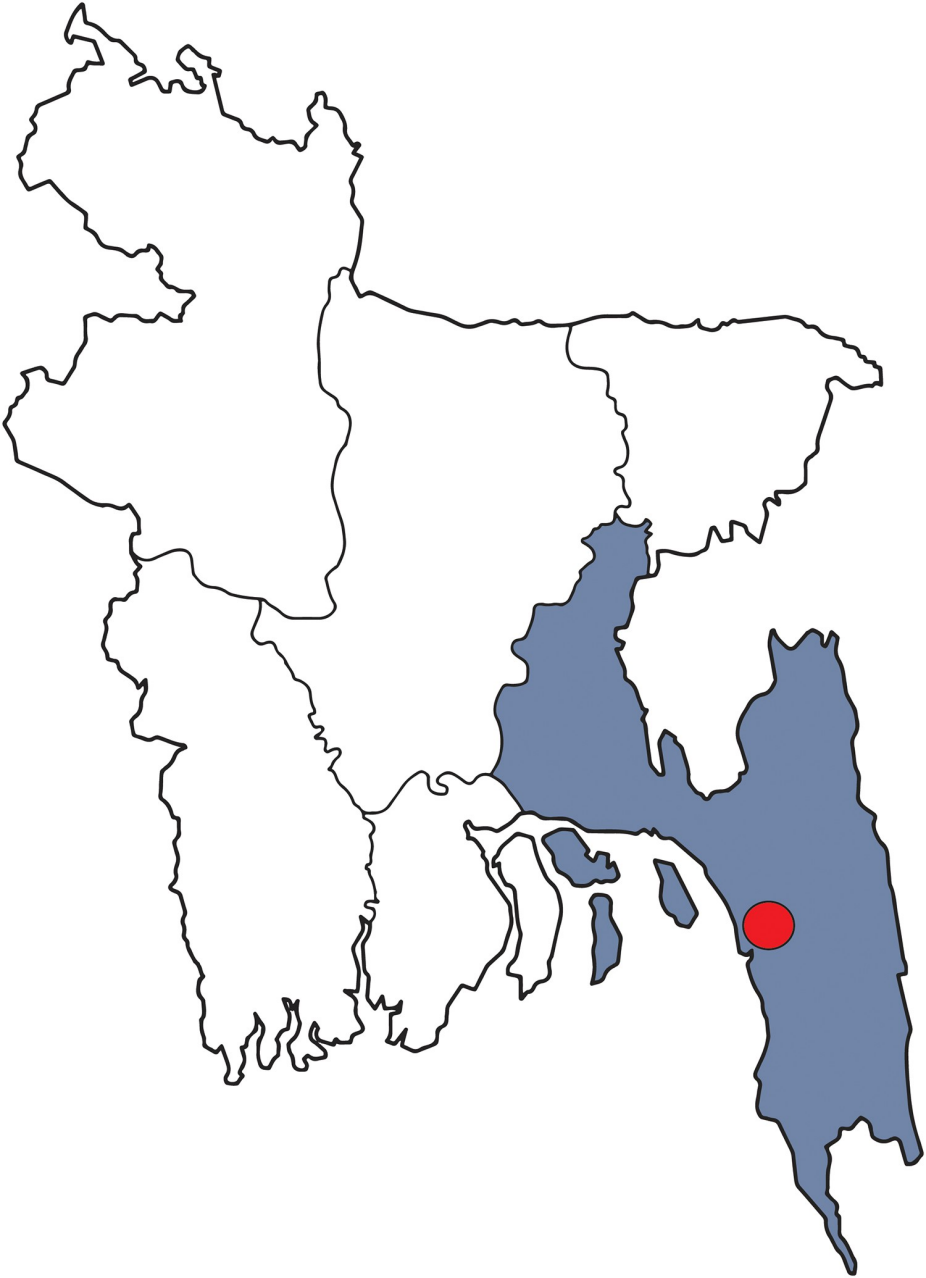
Geographical location of the studied population. The division Chattogram has been highlighted in blue and the location of the studied population is highlighted by the red bubble. The base layer of the map was collected from Wikimedia Commons (https://commons.wikimedia.org/wiki/File:Bangladesh_divisions_blank.png).

Data needed to be collected from minimum 385 individuals, which is the calculated sample size based on the above formula. In this study, the investigators collected data from 400 potential respondents. The criteria for including in our study were being a resident of Chattogram metropolitan area, above 18 years of age and voluntarily participation. The geographic location of the participants can be found in the Fig 1.

### 2.3. Measures

A pre-structured and self-reporting questionnaire was distributed that contained consent of participation, and questions regarding sociodemographic characteristics, knowledge, attitudes, and practices.

#### 2.3.1. Sociodemographic measures

Sociodemographic information of the participants such as gender, age, marital status, education level, occupation, monthly family income, employment status, and presence of children or elderly person in the family were collected for the study. Age group was classified into three classes: 18–35 years, 36–55 years, and >55 years. Monthly family income of the participants was categorized into five classes: <10000 Bangladeshi Taka (BDT), 10000–30000 BDT, 30000–50000 BDT, 50000–100000 BDT and >100000 BDT.

#### 2.3.2. Knowledge, attitudes and practices

To comprehend the degree of knowledge, attitudes, and practices of the participants along with their occupation differences, the questionnaire contains a total of 17 questions (comprising of 3 for knowledge, 4 for attitudes, and 10 for practices). The knowledge and attitudes questions were developed based on the objectives of the study and our target populations as we have already determined the key area of KAP that we want to investigate. On the other hand, the practices questions were generated based on the idea of immunity-boosting nutrients for SARS-CoV-2 that were either substantiated through scientific studies or hypothesized for having potential benefits [10–13].

Special emphasis was given during validation of questions related to knowledge, attitudes and practices. After completing the preliminary draft of the questionnaire, it was ratified and approved by two different actions. Initially, it was verified by an expert member where the expert opinion was considered. Finally, a statistical test was employed to measure the consistency of questions in the questionnaire. Cronbach’s alpha test for measuring the internal consistency of KAP questions was used. This statistic is a measure of reliability that is commonly used for demonstrating whether the tests and scales which were adopted in the research are fit for the desired purpose. The acceptable range of Cronbach’s alpha coefficient for its reliability is 0.45–0.98 [27]. In our study, the Cronbach’s alpha coefficient for knowledge, attitudes, and practices were 0.72, 0.62, and 0.79, respectively, and the overall Cronbach’s alpha of the questions was 0.75, which show the presence of acceptable internal consistency for each section of the questionnaire [27].

The knowledge section contained 3 questions and each of them had a probable response of “*Yes*”, and “*No*” (e.g., *Do you have idea about ‘Nutrition’*?). The correct answer (*Yes*) was then denoted by 1, and the incorrect answer (*No*) was denoted by 0. The total score for this category was calculated by summarizing the raw values of 3 individual questions. Therefore, the value for knowledge had a range between 0–3, and an overall higher score was the indication of “correct” knowledge. The cut off value for “correct” knowledge was ≥2.

The attitude section contained 4 questions and each question had a probable response of “*Yes*”, and “*No*” (e.g., *Do you wash fruits and vegetables brought from the market before eating them*?). The correct answer (*Yes/No*) was then denoted by 1, and the incorrect answer (Yes/*No*) was denoted by 0. After summarizing the raw values of the 4 individual questions, the total score for attitude ranged between 0–4, and an overall higher score was the indication of “favorable” attitude. The cut off value for “favorable” attitude was ≥3.

The practice section contained 10 questions and all of them had a possible response of “*Always*”, “*Often (4–5 days/week)*”, “*Sometimes (2–3 days/week)*”, “*Seldom (1 day/week)*”, and “*Never*” (e.g., *How often do you eat food that contains Vit-C (orange*, *tomato*, *pepper)*?). The desirable response (*Always/ Often/ Sometimes*) was then coded by 1, and the undesirable response (*Seldom/ Never*) was coded by 0. The summaries of the raw values for 10 practice questions ranged between 0–10, and an overall greater value was the indication of “good” practice. The cut off score for “good” practice was ≥9.

Finally, sensitivity analysis has been conducted by raising/lowering the cutoff score of KAP by one unit to highlight the robustness of the results (S1 Data). When the cutoff score of knowledge category was raised to one unit (≥3), the sensitivity of the result was 89.1%, and the sensitivity was 100% when the cutoff score was lowered to one unit (≥1). The sensitivity for attitude category was 64.9% when the cutoff score was increased to one unit (≥4), and the sensitivity was 100% when the score was decreased to one unit (≥2). In case of practice category, the sensitivity was 55.7% when the cutoff score was raised to one point (≥10) and the sensitivity was 100% when the cutoff score was lowered to one point (≥8). Thus, we proceeded with the selected cutoff values for KAP.

### 2.4. Statistical analysis

The statistical analysis of the data was performed by using Microsoft Excel 2021, IBM SPSS Statistics 26.0 (Chicago, IL, USA), and R for Windows v4.1.2 (R Core Team, Vienna, Austria). Initially, Microsoft Excel has been used to edit, sort and code the raw data. After that, the edited excel file was imported in SPSS where descriptive statistics (frequency measures, percentage etc.) and first order analysis (chi-square test) were conducted. Fisher’s exact test was selected instead of chi-square test when more than 20% cells had expected frequencies less than 5, and it was conducted on R for Windows. Then, binary logistic regression analysis was conducted on SPSS with 95% confidence interval for determining the significant association between dependent and independent qualitative variables. In the binary logistic regression, *Knowledge*, *Attitudes* and *Practices* were used as dependent qualitative variables, while sociodemographic variables named *Age*, *Gender*, *Marital status*, *Education*, *Occupation*, *Monthly family income*, *Are you employed during this quarantine*, and *Is there any child or elderly person in your family* were used as independent qualitative variables. Also, while using *Knowledge* as dependent variable, *Attitudes* and *Practices* were used as independent variables. Similarly, *Knowledge* and *Practices* were used as independent variables while using *Attitudes* as dependent variable, and *Knowledge* and *Attitudes* were used as independent variables while using *Practices* as dependent variable. Thus, the relationships between knowledge, attitudes, and practices could also be assessed (Table 6).

## 3. Results

The final analysis included 400 responders in total, of which 64.3% were male and the rest were female (35.8%), and the majority of them (82.5%) were between the ages of 18–35 years. More than half of the respondents were unmarried (69.5%). Most of them were students (62.7%), half of them had a bachelor’s level of education (50.0%), 35.5% of the responders’ family income was between 10,000 and 30,000 BDT per month, 50% of them were unemployed during the quarantine, and more than half of them had no children or elderly people in their family (Table 1).

**Table 1:** Sociodemographic characteristics of the respondents (N = 400).

Variables	Categories	n (%)
Gender	Male	257 (64.3)
	Female	143 (35.8)
Age (years)	18–35	330 (82.5)
	36–55	54 (13.5)
	>55	16 (4.0)
Marital status	Unmarried	278 (69.5)
	Married	118 (29.5)
	Divorced or widowed	4 (1.0)
Occupation	Student	251 (62.7)
	Government job	17 (4.3)
	Private job	26 (6.5)
	Business	33 (8.3)
	Service and sales	33 (8.3)
	Laborer	18 (4.5)
	Others	22 (5.5)
Education	Below SSC	50 (12.5)
	SSC	25 (6.3)
	HSC	66 (16.5)
	Bachelor’s degree	200 (50.0)
	Master’s degree or above	28 (7.0)
	Others	31 (7.8)
Monthly family income (BDT)	<10000	50 (12.5)
	10000–30000	142 (35.5)
	30000–50000	103 (25.8)
	50000–100000	79 (19.8)
	>100000	26 (6.5)
Are you employed during this quarantine?	Yes- Going to workplace	137 (34.3)
	Yes- Working from home	39 (9.8)
	No	202 (50.5)
	Others	22 (5.5)
Is there any child or elderly person in your family?	No	213 (53.3)
	Children less than 5 years	51 (12.8)
	Elders more than 65 years	68 (17.0)
	Both	68 (17.0)

In the overall assessment, 331 out 400 individuals had correct knowledge, 285 out of 400 had favorable attitude, and 176 out of 400 had good practices (S1 Data).

### 3.1. Knowledge

The distribution of responses from participants is shown for each knowledge question in Table 2 with occupational differences. For each of the knowledge questions, there were significant occupational differences. According to the results of this survey, 331 out of 400 (82.8%) respondents had correct knowledge regarding immunity boosting diets during COVID-19 (S1 Data). Most of them had an idea about nutrition (79.3%) and 20.8% of them had no idea regarding nutrition. Almost all of the responders (95.5%) agreed that "Strong immune system reduces the risk of death by COVID-19" (see Table 2). The proportion of those with greater correct knowledge was substantially more likely to be among (i) respondents aged between 18 to 35 years (89.7% in 18 to 35 years vs 53.7% in 36–55 and 37.5% in those aged more than 55 years, p-value <0.001) (ii) students (97.6% in students vs 51.5% in business, p-value <0.001) (iii) unmarried respondents (93.2% in unmarried vs 50% in those who are married p-value <0.001) (see Table 5).

**Table 2:** Knowledge and occupation differences of the respondents (N = 400).

Variable	Categories	Total n (%)	Student n (%)	Government job n (%)	Private job n (%)	Business n (%)	Service and Sales n (%)	Laborer n (%)	Others n (%)	*p*-value
Do you have idea about ’Nutrition’? ^a^	Yes	317 (79.3)	237 (94.4)	15 (88.2)	22 (84.6)	15 (45.5)	11 (33.3)	2 (11.1)	15 (68.2)	<0.001
	No	83 (20.8)	14 (5.6)	2 (11.8)	4 (15.4)	18 (54.5)	22 (66.7)	16 (88.9)	7 (31.8)	
Do you know the nutrients needed to strengthen our immune system? ^a^	Yes	314 (78.5)	236 (94.0)	16 (94.1)	21 (80.8)	16 (48.5)	11 (33.3)	1 (5.6)	13 (59.1)	<0.001
	No	86 (21.5)	15 (6.0)	1 (5.9)	5 (19.2)	17 (51.5)	22 (66.7)	17 (94.4)	9 (40.9)	
"Strong immune system reduces the risk of death by COVID-19"- Do you agree with this? ^a^	Yes	382 (95.5)	246 (98.0)	17 (100.0)	26 (100.0)	30 (90.9)	30 (90.9)	12 (66.7)	21 (95.5)	<0.001
	No	18 (4.5)	5 (2.0)	0 (0.0)	0 (0.0)	3 (9.1)	3 (9.1)	6 (33.3)	1 (4.5)	

^a^ Fisher’s Exact Test.

The respondents who were between 36–55 years were 0.699 times more likely to have correct knowledge on immunity boosting dietary behaviors than the respondents aging between 18 to 35 years (OR = 0.699, CI = 0.416–6.883, *p* = 0.463). The group having an HSC level of education had 23.168 times more correct knowledge than the group of responders having SSC level of education (OR = 23.168, CI = 2.616–205.186, *p* = 0.005) (see Table 6).

### 3.2. Attitudes

For each question focused on attitude, the distribution of responses from participants is presented in Table 3. We found that 285 out of 400 (71.3%) responders had a favorable attitude regarding immunity boosting diets during COVID-19 (S1 Data). This analysis also showed that all (100%) of the responders (n = 17) having a government job and 98.8% of the students (n = 248) wash fruits and vegetables brought from the market before eating them, and in total 98.5% of 400 responders do the same (Table 3). It was evident that most of the respondents (78%) don’t buy their food online. The response rates of "No" were significantly higher in students (66.5% vs. 87.9% in business, *p* value <0.001) to the item of the attitude section regarding "Do you often eat at restaurants?" (See Table 3).

**Table 3:** Attitudes and occupation differences of the respondents (N = 400).

Variable	Categories	Total n (%)	Student n (%)	Government job n (%)	Private job n (%)	Business n (%)	Service and Sales n (%)	Laborer n (%)	Others n (%)	*p*-value
Do you wash fruits and vegetables brought from the market before eating them? ^a^	Yes	394 (98.5)	248 (98.8)	17 (100.0)	26 (100.0)	33 (100.0)	32 (97.0)	17 (94.4)	21 (95.5)	0.288
	No	6 (1.5)	3 (1.2)	0 (0.0)	0 (0.0)	0 (0.0)	1 (3.0)	1 (5.6)	1 (4.5)	
Do you often eat at restaurants? ^b^	Yes	108 (27.0)	84 (33.5)	7 (41.2)	9 (34.6)	4 (12.1)	3 (9.1)	1 (5.6)	0 (0.0)	<0.001
	No	292 (73.0)	167 (66.5)	10 (58.8)	17 (65.4)	29 (87.9)	30 (90.9)	17 (94.4)	22 (100.0)	
Do you often purchase food online? ^a^	Yes	88 (22.0)	73 (29.1)	3 (17.6)	8 30.8)	3 (9.1)	1 (3.0)	0 (0.0)	0 (0.0)	<0.001
	No	312 (78.0)	178 (70.9)	14 (82.4)	18 (69.2)	30 (90.9)	32 (97.0)	18 (100.0)	22 (100.0)	
Do you often eat junk food (Fried fast food, salted snack food, soft drinks)? ^b^	Yes	188 (47.0)	128 (51.0)	4 (23.5)	17 (65.4)	13 (39.4)	17 (51.5)	4 (22.2)	5 (22.7)	0.003
	No	212 (53.0)	123 (49.0)	13 (76.5)	9 (34.6)	20 (60.6)	16 (48.5)	14 (77.8)	17 (77.3)	

^a^ Fisher’s Exact Test.

^b^ χ^2^ test.

The findings indicated that 66.7% of respondents (n = 110) whose age was between 18–35 years old had a favorable attitude (*p*-value <0.001). The proportion of those with more positive attitudes was substantially more likely to be (i) those who are employed (93.9% in service and sales, 87.9% in business, 82.4% in government jobs vs 62.5% in students, *p*-value <0.001), (ii) the older group (age > 35 years) (92.6% in aged 36–55 years, 93.8% in aged more than 55 years vs 66.7% in aged 18–35 years, *p*-value <0.001), (iii) responders who are married (n = 102) (86.4% in married group vs 64.7% in the unmarried group, *p*-value <0.001) (see Table 5).

Finally, in terms of characteristics associated with more favorable attitudes toward immunity boosting dietary behavior, we found being older (aged 36–55 years) vs younger (aged 18–20 years) significantly differed (OR = 1.692, CI = 0.416–6.883, *p* value = 0.463). Married respondents had 1.852 times more favorable attitudes than those who are unmarried (OR = 1.852, CI = 0.649–5.283, *p* value = 0.249). More favorable attitudes were seen in the responders who had government jobs, which is 6.048 times more than the students (OR = 6.048, CI = 1.074–34.051, *p* value = 0.041) (see Table 6).

### 3.3. Practices

For each question of practice, the distribution of responses from participants is presented in Table 4. This survey indicated that 176 respondents out of 400 (44.0%) had good practices regarding immunity boosting diets during COVID-19 (S1 Data). Most respondents (37.3%, n = 149) indicated that they sometimes (2–3 days per week) eat food that contains Vit-C (orange, tomato, pepper) and 41.0% of the respondents among them were students (n = 103, *p* value = 0.001). The response rate of "Sometimes (2–3 days/week)" was significantly higher in responders with private jobs (46.2% in private jobs vs 40.2% in students, *p* value = 0.022) to the item of practice section regarding "How often do you eat food that contains Vit-D (oily fish, egg yolk, liver) or expose yourself under sunlight?". Similarly, the "often (4–5 days/week)" response rate is significantly higher in Others (59.1% in others vs 51.5% in Business and 39.0% in students, *p* value = 0.004) (see Table 4).

**Table 4:** Practices and occupation differences of the respondents (N = 400).

Variable	Categories	Total n (%)	Student n (%)	Government job n (%)	Private job n (%)	Business n (%)	Service and Sales n (%)	Laborer n (%)	Others n (%)	*p*-value
How often do you eat food that contains Vit-C (orange, tomato, pepper)? ^a^	Always	104 (26.0)	45 (17.9)	6 (35.3)	10 (38.5)	19 (57.6)	9 (27.3)	5 (27.8)	10 (45.5)	0.001
	Often (4–5 days/week)	92 (23.0)	67 (26.7)	1 (5.9)	7 (26.9)	5 (15.2)	4 (12.1)	2 (11.1)	6 (27.3))	
	Sometimes (2–3 days/week)	149 (37.3)	103 (41.0)	6 (35.3)	9 (34.6)	7 (21.2)	14 (42.4)	6 (33.3)	4 (18.2)	
	Seldom (1 day/week)	51 (12.8)	34 (13.5)	4 (23.5)	0 (0.0)	2 (6.1)	5 (15.2)	4 (22.2)	2 (9.1)	
	Never	4 (1.0)	2 (0.8)	0 (0.0)	0 (0.0)	0 (0.0)	1 (3.0)	1 (5.6)	0 (0.0)	
How often do you eat food that contains Vit-D (oily fish, egg yolk, liver) or expose yourself under sunlight? ^a^	Always	54 (13.5)	28 (11.2)	4 (23.5)	6 (23.1)	5 (15.2)	3 (9.1)	6 (33.3)	2 (9.1)	0.022
	Often (4–5 days/week)	105 (26.3)	75 (29.9)	4 (23.5)	6 (23.1)	8 (24.2)	4 (12.1)	3 (16.7)	5 (22.7)	
	Sometimes (2–3 days/week)	163 (40.8)	101 (40.2)	7 (41.2)	12 (46.2)	13 (39.4)	12 (36.4)	5 (27.8)	13 (59.1)	
	Seldom (1 day/week)	70 (17.5)	44 (17.5)	2 (11.8)	2 (7.7)	4 (12.1)	13 (39.4)	4 (22.2)	1 (4.5)	
	Never	8 (2.0)	3 (1.2)	0 (0.0)	0 (0.0)	3 (9.1)	1 (3.0)	0 (0.0)	1 (4.5)	
How often do you eat food that contains Vit- E (plant-based oil, pumpkin, spinach, nuts)? ^a^	Always	29 (7.2)	18 (7.2)	1 (5.9)	2 (7.7)	2 (6.1)	2 (6.1)	4 (22.2)	0 (0.0)	0.745
	Often (4–5 days/week)	84 (21.0)	52 (20.7)	5 (29.4)	5 (19.2)	6 (18.2)	7 (21.2)	2 (11.1)	7 (31.8)	
	Sometimes (2–3 days/week)	156 (39.0)	94 (37.5)	7 (41.2)	15 (57.7)	11 (33.3)	12 (36.4)	8 (44.4)	9 (40.9)	
	Seldom (1 day/week)	120 (30.0)	80 (31.9)	4 (23.5)	4 (15.4)	13 (39.4)	10 (30.3)	4 (22.2)	5 (22.7)	
	Never	11 (2.8)	7 (2.8	0 (0.0)	0 (0.0)	1 (3.0)	2 (6.1)	0 (0.0)	1 (4.5)	
How often do you eat food that contains Vit-A (Beef liver, spinach, small fish, carrots, mango, papaya)? ^a^	Always	20 (5.0)	13 (5.2)	1 (5.9)	1 (3.8)	1 (3.0)	1 (3.0)	2 (11.1)	1 (4.5)	0.125
	Often (4–5 days/week)	70 (17.5)	50 (19.9)	2 (11.8)	5 (19.2)	5 (15.2)	2 (6.1)	2 (11.1)	4 (18.2)	
	Sometimes (2–3 days/week)	163 (40.8)	109 (43.4)	9 (52.9)	8 (30.8)	15 (45.5)	14 (42.4)	3 (16.7)	5 (22.7)	
	Seldom (1 day/week)	140 (35.0)	76 (30.3)	5 (29.4)	12 (46.2)	12 (36.4)	13 (39.4)	10 (55.6)	12 (54.5)	
	Never	7 (1.8)	3 (1.2)	0 (0.0)	0 (0.0)	0 (0.0)	3 (9.1)	1 (5.6)	0 (0.0)	
How often do you eat food that contains Vit-B6 (Fish, poultry, banana, nuts)? ^a^	Always	69 (17.3)	46 (18.3)	6 (35.3)	6 (23.1)	1 (3.0)	4 (12.1)	3 (16.7)	3 (13.6)	0.004
	Often (4–5 days/week)	156 (39.0)	98 (39.0)	7 (41.2)	11 (42.3)	17 (51.5)	7 (21.2)	3 (16.7)	13 (59.1)	
	Sometimes (2–3 days/week)	126 (31.5)	82 (32.7)	4 (23.5)	8 (30.8)	12 (36.4)	10 (30.3)	6 (33.3)	4 (18.2)	
	Seldom (1 day/week)	44 (11.0)	21 (8.4)	0 (0.0)	1 (3.8)	3 (9.1)	11 (33.3)	6 (33.3)	2 (9.1)	
	Never	5 (1.3)	4 (1.6)	0 (0.0)	0 (0.0)	0 (0.0)	1 (3.0)	0 (0.0)	0 (0.0)	
How often do you eat food that contains Vit-B9 (Green leafy veg, beans, eggs, lentils)? ^a^	Always	82 (20.5)	39 (15.5)	6 (35.3)	7 (26.9)	9 (27.3)	9 (27.3)	9 (50.0)	3 (13.6)	0.069
	Often (4–5 days/week)	148 (37.0)	95 (37. 8)	6 (35.3)	7 (26.9)	15 (45.5)	10 (30.3)	6 (33.3)	9 (40.9)	
	Sometimes (2–3 days/week)	128 (32.0)	87 (34.7)	4 (23.5)	10 (38.5)	8 (24.2)	12 (36.4)	2 (11.1)	5 (22.7)	
	Seldom (1 day/week)	39 (9.8)	28 (11.2)	0 (0.0)	2 (7.7)	1 (3.0)	2 (6.1)	1 (5.6)	5 (22.7)	
	Never	3 (0.8)	2 (0.8)	1 (5.9)	0 (0.0)	0 (0.0)	0 (0.0)	0 (0.0)	0 (0.0)	
How often do you eat food that contains Vit-B12 (Meat, Eggs and milk, cereals)? ^a^	Always	82 (20.5)	51 (20.3)	4 (23.5)	5 (19.2)	5 (15.2)	9 (27.3)	3 (16.7)	5 (22.7)	0.194
	Often (4–5 days/week)	148 (37.0)	93 (37.1)	5 (29.4)	13 (50.0)	17 (51.5)	5 (15.2)	7 (38.9)	8 (36.4)	
	Sometimes (2–3 days/week)	117 (29.3)	76 (30.3)	7 (41.2)	6 (23.1)	5 (15.2)	10 (30.3)	5 (27.8)	8 (36.4)	
	Seldom (1 day/week)	49 (12.3)	30 (12.0)	1 (5.9)	2 (7.7)	5 (15.2)	8 (24.2)	2 (11.1)	1 (4.5)	
	Never	4 (1.0)	1 (0.4)	0 (0.0)	0 (0.0)	1 (3.0)	1 (3.0)	1 (5.6)	0 (0.0)	
How often do you eat food that contains Zinc (Meat, Shellfish, legumes, poultry)? ^a^	Always	36 (9.0)	28 (11.2)	1 (5.9)	3 (11.5)	0 (0.0)	1 (3.0)	1 (5.6)	2 (9.1)	0.055
	Often (4–5 days/week)	90 (22.5)	64 (25.5)	4 (23.5)	4 (15.4)	7 (21.2)	2 (6.1)	3 (16.7)	6 (27.3)	
	Sometimes (2–3 days/week)	157 (39.3)	93 (37.1)	9 (52.9)	11 (42.3)	14 (42.4)	20 (60.6)	4 (22.2)	6 (27.3)	
	Seldom (1 day/week)	106 (26.5)	61 (24.3)	3 (17.6)	8 (30.8)	10 (30.3)	10 (30.3)	7 (38.9)	7 (31.8)	
	Never	11 (2.8)	5 (2.0)	0 (0.0)	0 (0.0)	2 (6.1)	0 (0.0)	3 (16.7)	1 (4.5)	
How often do you eat food that contains selenium (Nuts and seeds, Eggs, seafood, spinach, green peas)? ^a^	Always	22 (5.5)	13 (5.2)	1 (5.9)	2 (7.7)	2 (6.1)	1 (3.0)	2 (11.1)	1 (4.5)	0.259
	Often (4–5 days/week)	85 (21.3)	53 (21.1)	3 (17.6)	8 (30.8)	6 (18.2)	5 (15.2)	4 (22.2)	6 (27.3)	
	Sometimes (2–3 days/week)	158 (39.5)	101 (40.2)	9 (52.9)	8 (30.8)	20 (60.6)	13 (39.4)	2 (11.1)	5 (22.7)	
	Seldom (1 day/week)	123 (30.8)	76 (30.3)	4 (23.5)	8 (30.8)	5 (15.2)	12 (36.4)	9 (50.0)	9 (40.9)	
	Never	12 (3.0)	8 (3.2)	0 (0.0)	0 (0.0)	0 (0.0)	2 (6.1)	1 (5.6)	1 (4.5)	
How often do you eat food that contains Iron (Lean red meat, beans & lentils, Apple & banana)? ^a^	Always	17 (4.3)	12 (4.8)	1 (5.9)	2 (7.7)	0 (0.0)	0 (0.0)	2 (11.1)	0 (0.0)	0.089
	Often (4–5 days/week)	63 (15.8)	44 (17.5)	5 (29.4)	2 (7.7)	6 (18.2)	2 (6.1)	2 (11.1)	2 (9.1)	
	Sometimes (2–3 days/week)	196 (49.0)	116 (46.2)	10 (58.8)	17 (65.4)	19 (57.6)	14 (42.4)	6 (33.3)	14 (63.6)	
	Seldom (1 day/week)	113 (28.2)	71 (28.3)	1 (5.9)	5 (19.2)	8 (24.2)	16 (48.5)	6 (33.3)	6 (27.3)	
	Never	11 (2.8)	8 (3.2)	0 (0.0)	0 (0.0)	0 (0.0)	1 (3.0)	2 (11.1)	0 (0.0)	

^a^ Fisher’s Exact Test.

Furthermore, females had slightly more good practice towards immunity boosting dietary behavior than males (OR = 1.114, CI = 0.693–1.790. *p* value = 0.656) (see Table 6). The proportion of good practice was much higher in (i) Government job responder group (52.9% in government job vs 42.4% in business, *p* value = 0.002), (ii) females (50.3% in females vs 40.5% in males, *p* value = 0.056), (iii) responder group having education up to Master’s degree or above (57.1% in Master’s degree or above vs 24% in Below SSC, *p* value = 0.008), (iv) responders with higher family income (53.8% in >100000 vs 35.2% in 10000–30000, *p* value = 0.083) (see Table 5).

**Table 5:** Test of statistical significance of the variations in the respondents’ knowledge, attitudes, and practices on immunity boosting dietary behavior by their characteristics (N = 400).

Variables	Categories	Knowledge			Attitudes			Practices		
		Incorrect n (%)	Correct n (%)	*p*-value	Unfavorable n (%)	Favorable n (%)	*p*-value	Bad n (%)	Good n (%)	*p*-value
Age (years)	18–35	34 (10.3)	296 (89.7)	<0.001	110 (33.3)	220 (66.7)	<0.001	173 (52.4)	157 (47.6)	0.007
	36–55	25 (46.3)	29 (53.7)		4 (7.4)	50 (92.6)		39 (72.2)	15 (27.8)	
	>55	10 (62.5)	6 (37.5)		1 (6.3)	15 (93.8)		12 (75.0)	4 (25.0)	
Gender	Male	64 (24.9)	193 (75.1)	<0.001	66 (25.7)	191 (74.3)	0.069	153 (59.5)	104 (40.5)	0.056
	Female	5 (3.5)	138 (96.5)		49 (34.3)	94 (65.7)		71 (49.7)	72 (50.3)	
Marital status	Unmarried	19 (6.8)	259 (93.2)	<0.001	98 (35.3)	180 (64.7)	<0.001	147 (52.9)	131 (47.1)	0.049
	Married	48 (40.7)	70 (59.3)		16 (13.6)	102 (86.4)		76 (64.4)	42 (35.6)	
	Divorced or widowed	2 (50.0)	2 (50.0)		1 (25.0)	3 (75.0)		1 (25.0)	3 (75.0)	
Education	Below SSC	33 (66.0)	17 (34.0)	<0.001	3 (6.0)	47 (94.0)	<0.001	38 (76.0)	12 (24.0)	0.008
	SSC	7 (28.0)	18 (72.0)		5 (20.0)	20 (80.0)		18 (72.0)	7 (28.0)	
	HSC	2 (3.0)	64 (97.0)		23 (34.8)	43 (65.2)		32 (48.5)	34 (51.5)	
	Bachelor’s degree	5 (2.5)	195 (97.5)		66 (33.0)	134 (67.0)		105 (52.5)	95 (47.5)	
	Master’s degree or above	2 (7.1)	26 (92.9)		13 (46.4)	15 (53.6)		12 (42.9)	16 (57.1)	
	Others	20 (64.5)	11 (35.5)		5 (16.1)	26 (83.9)		19 (61.3)	12 (38.7)	
Occupation	Student	6 (2.4)	245 (97.6)	<0.001	94 (37.5)	157 (62.5)	<0.001	127 (50.6)	124 (49.4)	0.002
	Government job	1 (5.9)	16 (94.1)		3 (17.6)	14 (82.4)		8 (47.1)	9 (52.9)	
	Private job	4 (15.4)	22 (84.6)		10 (38.5)	16 (61.5)		14 (53.8)	12 (46.2)	
	Business	16 (48.5)	17 (51.5)		4 (12.1)	29 (87.9)		19 (57.6)	14 (42.4)	
	Service and sales	20 (60.6)	13 (39.4)		2 (6.1)	31 (93.9)		28 (84.8)	5 (15.2)	
	Laborer	16 (88.9)	2 (11.1)		1 (5.6)	17 (94.4)		15 (83.3)	3 (16.7)	
	Others	6 (27.3)	16 (72.7)		1 (4.5)	21 (95.5)		13 (59.1)	9 (40.9)	
Monthly family income (BDT)	<10000	15 (30.0)	35 (70.0)	<0.001	12 (24.0)	38 (76.0)	0.036	29 (58.0)	21 (42.0)	0.083
	10000–30000	38 (26.8)	104 (73.2)		31 (21.8)	111 (78.2)		92 (64.8)	50 (35.2)	
	30000–50000	12 (11.7)	91 (88.3)		37 (35.9)	66 (64.1)		52 (50.5)	51 (49.5)	
	50000–100000	3 (3.8)	76 (96.2)		23 (29.1)	56 (70.9)		39 (49.4)	40 (50.6)	
	>100000	1 (3.8)	25 (96.2)		12 (46.2)	14 (53.8)		12 (46.2)	14 (53.8)	
Are you employed during this quarantine?	Yes- Going to workplace	55 (40.1)	82 (59.9)	<0.001	28 (20.4)	109 (79.6)	0.048	92 (67.2)	45 (32.8)	0.012
	Yes- Working from home	1 (2.6)	38 (97.4)		15 (38.5)	24 (61.5)		21 (53.8)	18 (46.2)	
	No	11 (5.4)	191 (94.6)		64 (31.7)	138 (68.3)		101 (50.0)	101 (50.0)	
	Others	2 (9.1)	20 (90.9)		8 (36.4)	14 (63.6)		10 (45.5)	12 (54.5)	
Is there any child or elderly person in your family?	No	23 (10.8)	190 (89.2)	<0.001	63 (29.6)	150 (70.4)	0.946	117 (54.9)	96 (45.1)	0.732
	Children less than 5 years	11 (21.6)	40 (78.4)		13 (25.5)	38 (74.5)		29 (56.9)	22 (43.1)	
	Elders more than 65 years	22 (32.4)	46 (67.6)		19 (27.9)	49 (72.1)		42 (61.8)	26 (38.2)	
	Both	13 (19.1)	55 (80.9)		20 (29.4)	48 (70.6)		36 (52.9)	32 (47.1)	
Knowledge	Incorrect	69 (100.0)	0 (0.0)	-	6 (8.7)	63 (91.3)	<0.001	52 (75.4)	17 (24.6)	<0.001
	Correct	0 (0.0)	331 (100.0)		109 (32.9)	222 (67.1)		172 (52.0)	159 (48.0)	
Attitudes	Unfavorable	6 (5.2)	109 (94.8)	<0.001	115 (100.0)	0 (0.0)	-	57 (49.6)	58 (50.4)	0.100
	Favorable	63 (22.1)	222 (77.9)		0 (0.0)	285 (100.0)		167 (58.6)	118 (41.4)	
Practices	Bad	52 (23.2)	172 (76.8)	<0.001	57 (25.4)	167 (74.6)	0.100	224 (100.0)	0 (0.0)	-
	Good	17 (9.7)	159 (90.3)		58 (33.0)	118 (67.0)		0 (0.0)	176 (100.0)	

The sociodemographic factors of good practices were age (older vs younger: OR = 0.468, CI = 0.176–1.242.790. *p* value = 0.127), sex (female vs male: OR = 1.114, CI = 0.693–1.790. *p* value = 0.656), having higher education (HSC) vs SSC (HSC vs SSC: OR = 1.187, CI = 0.380–3.708, *p* value = 0.768). The findings show that responders with government jobs have 2.401 times more good practice towards immunity boosting dietary behavior than students (OR = 2.401, CI = 0.573–10.054, *p* value = 0.231). There is also an increase in good practice with the increase in family income (>100000 vs <10000: OR = 1.241, CI = 0.438–3.512, *p* value = 0.684) (see Table 6).

**Table 6:** Binary logistic regression analysis of the factors associated with respondents’ knowledge, attitudes, and practices on immunity boosting dietary behavior.

Variables	Categories	Knowledge	Attitudes	Practices
		OR, 95% CI, *p*	OR, 95% CI, *p*	OR, 95% CI, *p*
Age (years)	18–35	Ref.	Ref.	Ref.
	36–55	0.699, 0.189–2.578, 0.591	1.692, 0.416–6.883, 0.463	0.468, 0.176–1.242, 0.127
	>55	0.782, 0.105–5.832, 0.811	1.337, 0.119–14.999, 0.814	0.333, 0.073–1.514, 0.155
Gender	Male	Ref.	Ref.	Ref.
	Female	13.012, 1.534–110.390, 0.019	0.993, 0.594–1.661, 0.979	1.114, 0.693–1.790, 0.656
Marital status	Unmarried	Ref.	Ref.	Ref.
	Married	0.723, 0.170–3.073, 0.661	1.852, 0.649–5.283, 0.249	1.257, 0.523–3.023, 0.609
	Divorced or widowed	0.339, 0.005–22.035, 0.611	0.286, 0.014–5.722, 0.413	9.374, 0.746–117.836, 0.083
Education	Below SSC	Ref.	Ref.	Ref.
	SSC	2.097, 0.409–10.760, 0.375	0.387, 0.068–2.213, 0.286	0.454, 0.120–1.709, 0.243
	HSC	23.168, 2.616–205.186, 0.005	0.241, 0.051–1.145, 0.073	1.187, 0.380–3.708, 0.768
	Bachelor’s degree	10.939, 1.912–62.574, 0.007	0.369, 0.083–1.647, 0.192	0.949, 0.327–2.757, 0.924
	Master’s degree or above	5.083, 0.496–52.112, 0.171	0.119, 0.021–0.673, 0.016	1.162, 0.312–4.332, 0.823
	Others	0.700, 0.158–3.096, 0.638	0.450, 0.083–2.453, 0.356	1.639, 0.528–5.091, 0.393
Occupation	Student	Ref.	Ref.	Ref.
	Government job	1.650, 0.055–49.623, 0.773	6.048, 1.074–34.051, 0.041	2.401, 0.573–10.054, 0.231
	Private job	0.178, 0.015–2.133, 0.173	1.338, 0.372–4.805, 0.656	1.428, 0.450–4.534, 0.546
	Business	0.059, 0.005–0.751, 0.029	3.603, 0.714–18.191, 0.121	2.133, 0.588–7.732, 0.249
	Service and sales	0.249, 0.019–3.254, 0.289	3.955, 0.613–25.520, 0.148	0.502, 0.121–2.086, 0.343
	Laborer	0.032, 0.002–0.514, 0.015	4.134, 0.339–50.482, 0.266	0.441, 0.077–2.525, 0.358
	Others	0.025, 0.002–0.292, 0.003	9.398, 0.924–95.576, 0.058	0.857, 0.250–2.941, 0.806
Monthly family income (BDT)	<10000	Ref.	Ref.	Ref.
	10000–30000	2.994, 0.772–11.609, 0.113	1.072, 0.468–2.457, 0.869	0.831, 0.408–1.693, 0.610
	30000–50000	3.172, 0.711–14.165, 0.130	0.672, 0.290–1.555, 0.353	0.989, 0.473–2.068, 0.977
	50000–100000	37.610, 5.504–256.976, <0.001	0.845, 0.342–2.090, 0.715	1.204, 0.545–2.658, 0.646
	>100000	19.782, 1.231–317.971, 0.035	0.504, 0.162–1.568, 0.237	1.241, 0.438–3.512, 0.684
Are you employed during this quarantine?	Yes- Going to workplace	Ref.	Ref.	Ref.
	Yes- Working from home	3.388, 0.100–114.645, 0.497	1.662, 0.587–4.705, 0.339	1.449, 0.542–3.872, 0.460
	No	0.628, 0.059–6.671, 0.699	2.350, 0.981–5.628, 0.055	1.930, 0.838–4.441, 0.122
	Others	0.856, 0.035–20.976, 0.924	1.512, 0.417–5.480, 0.529	2.689, 0.817–8.849, 0.104
Is there any child or elderly person in your family?	No	Ref.	Ref.	Ref.
	Children less than 5 years	2.743, 0.713–10.546, 0.142	0.890, 0.387–2.046, 0.783	1.151, 0.566–2.339, 0.699
	Elders more than 65 years	0.588, 0.156–2.216, 0.433	0.807, 0.403–1.619, 0.547	0.898, 0.480–1.680, 0.736
	Both	0.356, 0.105–1.209, 0.098	0.880, 0.451–1.718, 0.709	1.141, 0.629–2.067, 0.665
Knowledge	Incorrect	-	Ref.	Ref.
	Correct	-	0.758, 0.234–2.456, 0.644	1.448, 0.601–3.488, 0.409
Attitudes	Unfavorable	Ref.	-	Ref.
	Favorable	0.765, 0.200–2.918, 0.695	-	0.915, 0.567–1.477, 0.717
Practices	Bad	Ref.	Ref.	-
	Good	2.073, 0.759–5.666, 0.155	0.905, 0.558–1.469, 0.687	-

## 4. Discussion

Coronavirus is currently a global danger that affects practically all countries. Individuals on their own are learning about COVID-19 through numerous means such as social media, television, print media and newspapers. Furthermore, the government is carrying out steps that they deem to be of the highest significance. The purpose of this study was to assess the degree of knowledge, attitudes, and practices of immunity-boosting dietary behavior as a COVID-19 preventative intervention among the residents of Chattogram Metropolitan Area (CMA), Bangladesh along with their occupational differences. The findings indicate a significant number of sociodemographic characteristics that are associated with KAP, and could be beneficial for developing preventive health education programs for emerging infectious illnesses. Our study found that approximately 79 in every 100 (79.3%) participants had adequate knowledge about nutrition.

From Table 5, we have found there is statistically significant relationship between knowledge differences and all the sociodemographic variables presented in our study, which are *age*, *gender*, *marital status*, *education*, *occupation*, *monthly family income*, *are you employed during this quarantine*, and *is there any child or elderly person in your family*. Also, knowledge of the population showed significant association with their attitudes and practices. However, in case of attitudes, the significant relationship was with *age*, *marital status*, *Education*, *occupation* and the knowledge. Finally, the practices of the participants had significant association with their *age*, *marital status*, *education*, *occupation*, *are you employed during this quarantine*, and their knowledge. In binary logistic regression (Table 6), the knowledge was significant for the females, having HSC or bachelor’s degree, being in the occupation of business, laborer or others, and having a monthly family income between 50000–100000 or >100000. The attitudes were significant with having a master’s degree or above, and a government job. However, the practices were not significant with any of the predictor variables. Previous studies in China found from multiple logistic regression that sociodemographic characteristics which were associated with more positive attitudes were being older age, having higher education, being employed, being in a joint family, having higher monthly family income, and implementing more frequent practices [28].

To our knowledge, there had been no previous KAP studies regarding immunity boosting dietary behavior among the populations of Bangladesh, let alone in Chattogram, especially during COVID-19. However, in a previous study regarding the use of herbals for immunity boosting in Bangladesh, a substantial proportion of individuals (57.6 percent) reported of using herbal foods or products to reduce their risk of COVID-19 infection. About 71% of them drank hot tea (both regular and herbal teas); while 56.5 percent used ginger, black seed, honey, and clove combined; and 32.8 percent, 30 percent, and 28.8 percent of them used ginger, black seed, honey, and clove, respectively in individual. These plants were consumed either alone or in conjunction with tea or hot water. Being female, young (18–29 years), married, and fearful of the pandemic were all factors connected with using herbal foods/products. Participants with a graduate degree were also more likely than those with a secondary degree or below to use preventative herbal foods/products [29]. Nevertheless, in our study, we focused on the use of immunity boosting nutrients (or nutrient rich foods), which were either previously substantiated or hypothesized to be potential for immune boosting by relevant scientific studies.

In a study on Jordanian adolescents, over half of the teens reported eating vitamin-C rich foods believing it enhances their defense against coronavirus [30]. Although there is no strong evidence that vitamin-C can protect against Coronavirus, it is sensible to add this nutrient in the dietary management of COVID-19 considering its antiviral and immunity boosting properties [31,32]. Another KAP study toward COVID-19 on Lebanese populations indicated that 76.1 percent of the people increased their consumption of fruits and vegetables, 59.5% took vitamin-C supplements, 56.1% exercised on a daily basis, and 47.6% avoided fast food in an attempt to boost their immunity and thus reduce their risk of becoming infected with the virus. These lifestyle changes are also beneficial in avoiding the majority of other illnesses, including diabetes and cardiovascular disease [33]. In our study, we have included all the immunity boosting nutrients and their sources for identifying whether the populations of CMA have good practices towards the consumption of those nutrients. Our study revealed that 56.0% of the populations of CMA had bad practices regarding immunity boosting diet despite having correct knowledge and favorable attitude among 82.8% and 71.3% of the populations, respectively. Correct knowledge and favorable attitudes may have developed among the populations due to mass awareness building guidelines during COVID-19 through different media and internet from the government as well as various international organizations. Bad practices were greatly seen among the people with educational qualifications of up to SSC (72.0%) or below SSC (76.0%), monthly family income of 10000–30000 BDT (64.8%), and being a laborer (83.3%) or having an occupation of service and sales (84.8%), hence these sociodemographic factors could play key roles regarding their bad practices of immunity boosting diet as some of these nutrient-rich foods are costly and people of all classes cannot afford those. There has been a recent KAP study regarding dietary supplementation among Lebanese population which also showed significant reduction in practice of taking dietary supplements, such as vitamins and minerals among the populations during the COVID-19 pandemic as compared to before the commencement of pandemic [34]. In another recent study on Indians staying in different countries showed that participants of all 4 divided regions had overall knowledge regarding immuno-nutrition, however the attitudes and practices differed by regions. For instance, decrease in practices of consuming immunity boosting nutrients were seen in Indians from East Africa who reported that 98.3% of them had knowledge about immunity boosting characteristics of vitamin C, yet only 47.5% had actual practice of taking the supplement [35]. The overall gaps in the practices despite having correct knowledge or favorable attitudes should be minimized by taking proper steps while considering the sociodemographic factors identified through this study.

## 5. Limitations

This study has some limitations. Firstly, it was a cross-sectional study, hence causal inferences could have not been made. Secondly, online reporting of the questionnaire may have several biases, although half of the study population was added through in-person interviews. Specifically, the online-based participants were mostly students, hence most of our participants were aged between 18–35 years old. There were also chances of response bias during online reporting. Thirdly, during the in-person interviews, most females declined to take part in the study, which resulted in a larger number of males in the study. Also, there could be presence of social desirability bias/reporting bias as people may not say what they truly believe but rather what they ought to do. Fourthly, the samples of the study were collected only from the populated or major sites of CMA, thus it caused exclusion of some smaller areas. Moreover, the use of purposive sampling may have unintentionally caused some selection bias or interviewer bias. Finally, we have used limited numbers of questions for measuring the degree of knowledge, attitudes, and practices, specifically three questions for knowledge and four questions for attitudes as we wanted to focus more on the practices category that included ten questions. Thus, the questionnaire itself has some limitations. Lastly, this KAP study regarding immunity-boosting diet did not construct the multi-dimensional measures.

## 6. Conclusion

The findings suggest that during the lockdown period when higher transmission of COVID-19 was occurring, the populations of CMA showed substantive differences in their knowledge, attitudes, and practices regarding their dietary behavior. The findings of this study also indicate the necessity of effectual and tailored educational programs for ameliorating correct knowledge, favorable attitudes, and good practices regarding immunity-boosting dietary behavior during and even after COVID-19 among the populations. However, the result showed the presence of bad or unhealthy practices among the populations despite having correct knowledge and favorable attitudes, thus emphasis on this category should be given while making public health campaigns or training programs. Moreover, other population characteristics, such as gender differences, education, monthly family income, occupation etc. should be kept in consideration during these programs.

## Supporting information

S1 Data Data from survey and primary analysis. (XLSX)
